# Synthesis in the glycosciences II

**DOI:** 10.3762/bjoc.8.45

**Published:** 2012-03-20

**Authors:** Thisbe K Lindhorst

**Affiliations:** 1 Otto Diels Institute of Organic Chemistry, Christiana Albertina University of Kiel, Otto-Hahn-Platz 3/4, 24098 Kiel, Germany

In 2010 the Beilstein Journal of Organic Chemistry launched a Thematic Series entitled “Synthesis in the glycosciences” with 14 contributions from renowned research groups in carbohydrate chemistry [1–14]. This series impressively demonstrated the power and the passion, the creativity and the cleverness of modern synthetic glycoscience. But this success is on a continuing journey. Thus, “Synthesis in the glycosciences II” has been started, and it gives me pleasure to introduce you to this Thematic Series today.

Here I have decided not to limit the field of the glycosciences to just synthetic chemistry but to let the contributions strike out as far as this intriguing field of science allows us go. It is interesting to see that, following this editorial approach, the diversity of titles reflects the diversity of carbohydrate chemistry and biology. There is no other class of natural products with a comparable potential for molecular diversity. There are no other biopolymers so difficult to assemble as the oligosaccharides. And there is no other cell organelle that is less understood in its detailed biochemical function and the meaning of its supramolecular identity than the glycocalyx. Hence, the field of “glycomics” may comprise a rather infant field of the “omics era”, but it is certainly one of the most exciting and promising divisions of modern chemistry.

The many different works contributed to this Thematic Series impressively demonstrate the variety in the glycosciences. Fantasy and imagination have led to novel glycoconjugate architectures and glycobiological experiments. Expertise and rational planning have allowed the utilization of carbohydrates in stereoselective synthesis and the employment of enzymes in oligosaccharide synthesis. Analytical and pharmacological knowhow have disclosed polysaccharide and glycoconjugate structures, their biological effects and their potential as carbohydrate drugs. Boldness and interdisciplinary communication have opened the field for many medical applications benefitting human health, such as in tumor diagnosis, tumor treatment and vaccination.

One of the objectives of the glycosciences is to unravel the secrets of carbohydrate biology in living systems. For this endeavour, chemical, biological and physical concepts and experiments have been equally important and equally challenging. In addition computer-aided methods, such as molecular dynamics studies of carbohydrates and glycoconjugates, have added valuable information about the characteristics of carbohydrate assembly. Furthermore, the field of glycobioinformatics has been recently invented and has started to assist researchers in managing an unprecedented number of structures and amount of biological information [15].

In this second captivating Thematic Series, the pride of the glycosciences is the answer to prejudice about a field which has been underestimated for most of the 20^th^ century. Many facets of the comprehensive field of the glycosciences will be found in this Thematic Series. It has been a pleasure to orchestrate this sound, and resounding, collection. I am thankful to the authors who have earned the credits of this volume and to the entire Beilstein editorial team who have been tremendously positive, enthusiastic and serviceable. The secret of achievement is to hold a picture of a successful outcome in the mind. The outcome of this successful Thematic Series is a survey giving a representative example of “sweet” diversity.

Thisbe K. Lindhorst

Kiel, March 2012
